# Delirium Detection Using Point-of-Care Single-Channel EEG: A Pilot Study

**DOI:** 10.1093/geroni/igaf122.3744

**Published:** 2025-12-31

**Authors:** Rebecca Heinze, Tage Runkle, Yvonne Larson-Smith, Emma Behnken, Gen Shinozaki, Allyson Palmer

**Affiliations:** Macalaster College, Rosemount, Minnesota, United States; Mayo Clinic, Rochester, Minnesota, United States; Mayo Clinic, Rochester, Minnesota, United States; Mayo Clinic, Rochester, Minnesota, United States; Stanford University, Palo Alto, California, United States; Mayo Clinic, Rochester, Minnesota, United States

## Abstract

Delirium is a common yet underdiagnosed medical emergency involving acute deficits in cognition and attention. It affects over 20% of hospitalized patients and up to 80% of ICU patients, with increased prevalence in older adults. Delirium is associated with increased mortality, prolonged hospitalization, long-term cognitive decline, and substantial healthcare costs. However, current screening tools are subjective and difficult to implement consistently. Traditional EEGs show diagnostic utility; however, they are costly and require specialized interpretation. We conducted a prospective pilot study evaluating a point-of-care, single-channel EEG device for delirium detection in hospitalized older adults. EEG spectra were utilized to calculate a ‘bispectral EEG’ (BSEEG) score reflecting the ratio of slow to fast brain waves. Twenty-two postoperative patients (mean age 75; 59.09% female) were assessed daily for up to seven days using both the BSEEG and the gold standard 3-Minute Diagnostic Interview for Confusion Assessment Method (3D-CAM). ROC analysis was utilized to determine an optimal threshold BSEEG score to indicate delirium positivity. BSEEG scores were significantly higher in the 3D-CAM-positive group than in the 3D-CAM-negative group (1.60 and 1.45, respectively; p < 0.05). A BSEEG threshold of 1.5 demonstrated 77.78% sensitivity and 56.10% specificity for delirium. In contrast, route clinical screenings (Delirium Triage Screen and Brief CAM) showed 0.00% sensitivity and 96.30% specificity, and failed to detect all true delirium cases. Although further validation in larger cohorts is needed, these findings suggest that the point-of-care single-channel EEG is a promising, non-invasive screening tool for delirium screening, particularly in fast-paced clinical settings.

